# Intravenous Paracetamol for Postoperative Analgesia in Laparoscopic Cholecystectomy

**DOI:** 10.5812/aapm.9880

**Published:** 2013-07-01

**Authors:** Sayed Mohamadreza Gousheh, Sholeh Nesioonpour, Fatemeh Javaher foroosh, Reza Akhondzadeh, Sayed Ali Sahafi, Zeinab Alizadeh

**Affiliations:** 1 Department of Anesthesiology, Ahvaz Jundishapur University of Medical Sciences, Ahvaz, Iran

**Keywords:** Acetaminophen, Pain, Cholecystectomy, Laparoscopic, Analgesics, Opioid

## Abstract

**Background:**

Although opioids are the main choice for acute postoperative pain control, many side effects have been reported for them. NSAIDs and paracetamol have been used extensively as alternatives, and it seems that they are more effective for minor to moderate pain control postoperatively when have been used alone or in combination with opioids. As laparoscopic cholecystectomy poses moderate pain postoperatively, this study was planned to assess whether paracetamol is able to provide effective analgesia as a sole analgesic at least in the first few hours post operatively.

**Objectives:**

We evaluated the effect of intravenous Paracetamol on postoperative pain in patients undergoing laparoscopic cholecystectomy.

**Patients and Methods:**

This is a randomized double- blind clinical trial study. 30 patients ASA class I, aged 18 to 50 years, candidate for laparoscopic cholecystectomy were recruited, and randomly divided into two equal groups. Group A (paracetamol group) received 1 gr paracetamol and group B received placebo ten minutes after the induction of anesthesia. 0.1 mg/Kg Morphine was administered intravenously based on patients compliant and pain score >3. Pain score and the opioids consumption were recorded in the first six hours postoperative. Patient's pain was measured by the VAS (Visual Analog Scale).

**Results:**

The pain score was lower in group A (P= 0.01), but the morphine consumption showed no significant difference between the groups (P= 0.24) during the first 6 hours postoperatively.

**Conclusions:**

Although paracetamol (1gr) has caused a better pain relief quality but it is not a suitable analgesic for moderate pain control in acute phase after surgery alone.

## 1. Background

Pain is an unpleasant sensory and emotional experience associated with actual or potential tissue damage. Undergoing treatment (such as surgical procedures) may result in the occurrence of postoperative pain, and this triggers biochemical and physiological stress responses (1). Pain is a major public health issue throughout the world and represents a major clinical, social, and economic problem (2). Postsurgical pain is normally perceived as nociceptive pain. Surgical trauma has been known to induce central and peripheral sensitization and hyperalgesia, which in untreated cases could lead to chronic postoperative pain after surgery (3). Proper pain management, particularly postoperative pain management, is a major concern for clinicians as well as for patients undergoing surgery. Patients commonly enquire about the level of pain they may experience after an operation. Postoperative pain not only affects the patients’ operative outcome, well-being, and satisfaction from medical care, but also directly affects the development of tachycardia, hyperventilation, decreases in alveolar ventilation, transition to chronic pain, poor wound healing, and insomnia, which in turn may impact the operative outcomes (4, 5). Individual variations in the response to pain are influenced by the genetic makeup, cultural background, age, and gender (6). The practice of modern anesthesiology has been developed from intraoperative period into perioperative period. Postoperative pain management is one of the most important components of adequate postsurgical patient’s care (7). Pain during and after surgery can lead to sensitization and consequently oversensitivity to pain, it can also transform postoperative acute pain into chronic pain (8). Effective postoperative pain control is important, especially with the initiation of physiotherapy and early ambulation, which hastens recovery and reduces hospital length of stay (9). The use of opioid drugs for the pain control during and after surgery is a common procedure in anesthesia (10). However, the use of these medications is associated with side effects such as nausea, vomiting, sedation, and respiratory depression. Prescribed method for reducing and minimizing opioid side effects is concomitant administration of a nonopioid analgesic (11). The decision to mix drugs should not be made without the knowledge of their compatibility. Incompatibility problems are more likely to arise when small concentrated volumes are mixed in a syringe rather than in the large volume of infusion bag (12). Some of these medications are nonsteroidal anti-inflammatory drugs, including aspirin and Acetaminophen (Paracetamol). Primary mechanism of these analgesic drugs is to inhibit the cyclooxygenase and prostaglandin synthesis which is considered as an important environmental factor in the prevention of hypersensitivity and pain (10). To date, at least two types of cyclooxygenase (COX) have been identified: Cyclooxygenase1 (COX-1) which is involved in platelet aggregation, hemostasis, and protects the gastric mucosa, and cyclooxygenase 2 (COX-2) which is effective in pain, inflammation, and fever. Recently discovered COX 3 (COX-3) has been proposed as a central mechanism for the analgesic effect of acetaminophen (13). Nonsteroidal anti-inflammatory drugs are usually effective for mild to moderate pain control. The effectiveness of these drugs has been identified as opioids adjuvant for moderate to severe pain. Recent studies have known that nonsteroidal anti-inflammatory drugs are effective for pain control rather alone or combined with opioids, which are more than what has been assumed so far (14, 15). While Paracetamol has been used for pain control after surgery (16), the use of this drug after induction of anesthesia for the postoperative pain control is the original point of this research that determined:

« Whether this drug is effective on opioid consumption in the pain control after surgery »

## 2. Objectives

Laparoscopic cholecystectomy has become the treatment of choice for most patients with symptomatic cholelithiasis (17). We evaluated the effect of intravenous Paracetamol on postoperative pain and opioid consumption in patients undergoing laparoscopic cholecystectomy.

## 3. Patients and Methods

In this double-blinded, prospective, randomized placebo- controlled trial, after approving by Ahvaz Jundishapur University of Medical Sciences (AJUMS) Ethical Committee, 30 patients of ASA class I, aged between 18 and 50 years, and scheduled for elective laparoscopic cholecystectomy under general anesthesia were recruited, and randomly divided into two equal groups in the specified time period. Simple random sampling was performed. Exclusion criteria contained the presence of: 1 - operation for less than an hour or greater than three hours 2- intraoperative bleeding greater than 6 cc / kg of body weight 3 - liver or kidney disease, 4 - patients with opioid or alcohol addiction 5 – patients' reluctance to participate in the study. The severity of pain was documented based on the VAS. The VAS is a standard tool like a 10 cm ruler including 10 numbers beginning from 0 (no pain), and ending at 10 (the most severe pain). The patient was asked to select a number based on the severity of pain he or she feels. Intravenous Paracetamol (Proparacetamol) was formulated for using during the anesthesia or immediately after it. However, other formulations have no high capability. Proparacetamol is rapidly hydrolyzed by plasma esterases to form paracetamol, as if a 1000 mg of Proparacetamol can produce 500 mg Paracetamol. Parastylaminofenol is the precise chemical formulation of Paracetamol. Through intravenous administration, the onset of effect is about half an hour, approximately one-hour half-life, and 6-8 hours effectiveness. The maximum recommended dose for adults is 4 grams in twenty-four hours. In this study, Paracetamol was provided by the COBEL DAROU pharmaceutical company. Patients were divided into two groups of A and B; and they did not receive any premedication drug before entering the operating room. After the placement of routine monitoring in all patients, including Electrocardiomyography and Pulseoximetry, noninvasive sphygmomanometer, and capnography, they received 5 cc / kg Ringer serum. Both groups were anesthetized with midazolam 0.03 mg/kg , Propofol 2mg/kg , Remifentanil 1 μg / kg over a minute, and Atracurium 0.5 mg/kg, and intubated at minimum possible time, and connected to a ventilator with % 50 O2, % 50 N2O after ensuring that the tube is in the trachea. Respiratory parameters were adjusted during the operation; so that oxygen saturation, end-expiratory carbon dioxide, and end expiratory volume were 96 -100%, 35-45 mmHg, and 10 ml per kg of body weight, respectively. Both cases and controls were the same as above. During the operation, patients received 0.1 μg/kg/min Remifentanil intravenous infusion and Isoflurane 1 MAC. In the treatment group, intravenous Paracetamol was injected 10 minutes after the induction of anesthesia with a bolus over 15 minutes (1gr Paracetamol in 100ml of 0.9 % NaCl). Intravenous infusion of Remifentanil (0.05 μg / kg / min or less) was added based on changes in blood pressure and heart rate more than 10% of base. In the control group, 100 cc 0.9 % NaCl was injected with a bolus over 15 minutes as placebo. During the operation, a dose of Intravenous Atracurium 0.2 mg/kg was repeated in both groups for every 30 minutes. Higher maintenance doses of Isoflurane were administered for increasing the depth of anesthesia, which were continuously controlled with the BIS (Bispectral index). Remifentanil was reduced (to half - dose) in both groups, on the extubation time for analgesia. All the operations were performed by the same surgeon. Laparoscopy was initiated after 15 minutes of anesthesia induction, and gas pressure was 13-15 mmHg all over the operation. At the end of the operation the neuromuscular blocking action was reversed by using atropine 0.02 mg/kg and 0.05 mg/kg neostigmine. At the end, the patients were extubated and transferred to the post anesthetic care unit. In the recovery room, oxygen 5 Lit / min with simple mask was administered, and patients were monitored by pulse oxymetry. The patients were maintained in recovery at least an hour. Postoperative pain was determined by the VAS for up to six-hour intervals: during extubation, 15 minutes, 30 minutes, one hour, two hours, three hours, four hours, five hours, and six hours after the extubation, and if the Visual Analog Scale (VAS) was more than 3, 0.1mg/kg morphine was administered intravenously. Total intravenous morphine consumption after the operation, the VAS and the side effects such as nausea, vomiting, laryngospasm and sedation were recorded at the time. Persons responsible for medication and follow-up in patients during and after the operations were unaware of the type of primary drug administered to the patient. All data were analyzed using the SPSS for Windows (version 12.0). The results of this study were collected and analyzed by independent sample t- test and Chi-square test. The significance level was set to P ≤ 0.05.

## 4. Results

Demographic data in the study including mean age, weight, and duration of operation in the two groups showed no statistically significant difference Table 1. The results of the variables are shown in Table 2 which indicates:

1)No significant difference for intravenous morphine consumption for the pain control (P = 0.24).

2)No significant difference for the first morphine requirement (P = 0.698).

**Figure 1.**
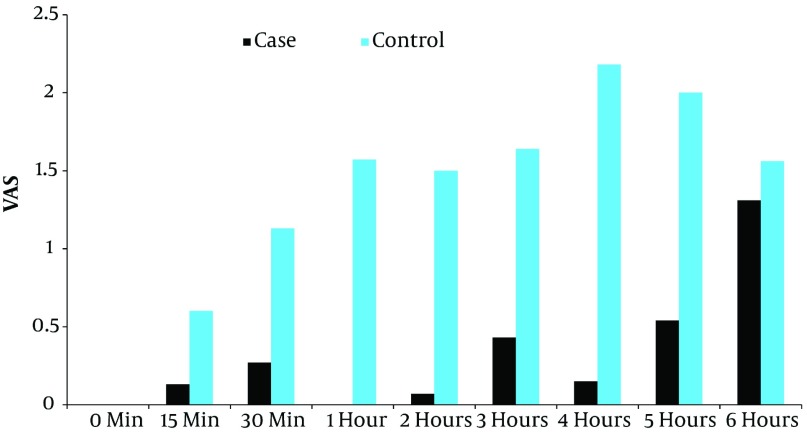
Comparison of the Two Groups in Mean VAS Outline

Comparison of the two groups in mean VAS in Figure 1 indicates that the difference in the VAS up to five hours after the operation was significant (P = 0. 01).

**Table 1.** Demographic Data of Patients

Groups	No.	Sex		Weight, kg, Mean ± SD	Age, y, Mean ± SD	Duration of the operation, Mean ± SD
		Male, %	Female, %			
Group	15	0	100	11.3 ± 73	31.6 ± 8.5	108.7 ± 17.1
Controls	15	6.7	3.93	69.9 ± 12.7	6.9 ± 35.9	21.6 ± 106
P value		1		0.048	0.014	0.711

**Table 2.** Values of Opioid Prescribed for Patients for up to 6 Hours After the Operation (Morphine)

Variable	Case, Mean ± SD	Control, Mean ± SD	P value	Significant differences
6 h of Morphine consumption	1.3 ± 2.8	2.7 ± 3.6	0. 24	No
1 time morphine	0.63 ± 1.67	0.87 ± 1.5	0.698	No

## 5. Discussion

In a study conducted in 2010 by Memis and colleagues, they reviewed the effect of intravenous Paracetamol in reducing opioid consumption, time of extubation, and opioid side effects in intubated patients admitted to the ICU. Ultimately, they concluded that intravenous Paracetamol reduces opioid consumption, the extubation time, and opioid side effects, such as nausea, vomiting, and itching (18). In this study, the same as our study, Paracetamol used for postoperative pain control had significant difference in the pain control between the groups, but unlike our study was also effective in reducing opioid consumption. In a study by Brodner and colleagues on patients undergoing mild to moderate surgery with general anesthesia, it was concluded that Paracetamol and other nonopioid analgesics have a similar effect (19). Postoperative pain was reduced with non-narcotic medication in comparison to placebo with no effect on pain control. Furthermore, in our study, the effect of Paracetamol was significant in the pain control. In a study conducted in 2009 by Samson and colleagues, efficacy and pharmacokinetics of intravenous Paracetamol were studied in the ICU patients, and found that after administration of 1 gr intravenous paracetamol four times a day, the optimal therapeutic dose is not available (20). In our study, the use of Paracetamol significantly reduced pain, but it had no effect on the opioid consumption. In another study in 2008 that was performed by Cakan and colleagues, intravenous Paracetamol improved the quality of postoperative analgesia but did not decrease the consumption of opioids (21). In our study, pain also was significantly lower than the control group with administration of 1 gr paracetamol, but there was no significant reduction on the opioid consumption. In a study conducted in 2007 by Borisov DB and colleagues using 1 g Paracetamol before abdominal surgery with epidural anesthesia, , did not reduce the pain and consumption of analgesics (22). In our study, also similar results were obtained. According to the results, we found that the intravenous administration of 1 gr Paracetamol immediately after the induction of anesthesia had no effects on opioid consumption between the two groups after laparoscopic cholecystectomy. In fact, administration of intravenous Paracetamol 1 gr immediately after the induction of anesthesia did not cause a decrease in the amount of opioid (morphine) consumption for the pain control after laparoscopic cholecystectomy (P = 0. 24). Because some studies have indicated the effects of anesthesia and surgery on Paracetamol pharmacokinetic and the time of peak plasma concentration, and these changes are different in patients who are undergoing surgery and anesthesia and in patients who are not undergoing surgery and anesthesia (23), noneffective intravenous 1 gr Paracetamol in postoperative opioid consumption reduction is justified to relieve pain. Generally, it can be seen that the administration of intravenous 1 gr Paracetamol after the induction of anesthesia, can be used as a complementary therapy for the pain control up to five hours after laparoscopic cholecystectomy, but opioid (morphine) consumption for the postoperative pain control is not resolved. Another study recommended a higher dose of Paracetamol for the pain control and reduction of the side effects of opioids after laparoscopic cholecystectomy .Moreover, the use of opioid medications along with 1 gr of intravenous Paracetamol is recommended for better pain control.
